# Hypermethylation of *BRM* promoter plays oncogenic roles in development of clear cell renal cell carcinoma

**DOI:** 10.7150/jca.30098

**Published:** 2019-08-28

**Authors:** Ru Fang, Qiuyuan Xia, Jing Sun, Hao Zha ng, Yan Liang, Xiaotong Wang, Xuan Wang, Henghui Ma, Xiaojun Zhou, Yang Cheng, Qiu Rao

**Affiliations:** 1 Department of Pathology, Jinling Hospital, Nanjing University School of Medicine, Nanjing, 210002, Jiangsu, China; 2 Center for Health Management, Geriatric Hospital of Nanjing Medical University, Nanjing, 210009, China;; 3 Department of Medical Oncology, the First Affiliated Hospital of Nanjing Medical University, Nanjing, 210029, China.; 4 Sir Run Run Shaw Hospital Affiliated to Nanjing Medical University, Nanjing, 210029, China.

**Keywords:** *BRM*, clear cell renal cell carcinoma, methylation, copy number variation, proliferation, apoptosis.

## Abstract

Although the inactivation of *BRM* plays oncogenic roles in tumorigenesis, regulation mechanism is rarely studied in clear cell renal cell carcinoma (RCC). Thus, we aimed to investigate the mechanism of *BRM* inactivation and explore the tumor suppressing roles of *BRM* in the development of clear cell RCC. We verified that hypermethylation of the *BRM* promoter was correlated with decreased expression of *BRM* by multi-omics analysis based on the TCGA database. This result was further confirmed in our own tumor tissues. Moreover, *BRM* inhibited the ability of proliferation and invasion of RCC cells* in vitro*. Consistent with this, *BRM* overexpressing virtually inhibited the xenograft tumor growth of ACHN cells *in vivo*. Finally we found that *BRM* promoted cell apoptosis and cellular cycle arrest in G2/M. In conclusion, our study confirms that the hypermethylation of *BRM* promoters plays oncogenic roles by the transcription inhibition of *BRM* in RCC, and the tumor suppressor gene *BRM* inhibits RCC cell vitality *in vitro* and* in vivo.*

## Introduction

Renal cell carcinoma (RCC), a common human malignant tumor in the genitourinary system, includes a variety of different pathological subtypes. The most common subtype is clear cell RCC, which accounts for approximately 80% of RCCs 1, 2. The pathogenesis of clear cell RCC has been linked to inactivation of the von Hippel-Lindau (*VHL*) tumor suppressor gene at chromosome 3p. However, it was also reported that simple inactivation of the *VHL* gene does not directly lead to tumorigenesis. These results indicate that *VHL* gene mutations are early event in the development of RCC, and the development of RCC involves other oncogenic factors, including somatic mutations, copy number variations and epigenetic silencing of tumor suppressor genes 2, 3.

The yeast mating type SWI/SNF (switch/sucrose nonfermenting) complex is a chromatin remodeling complex. Its core members include *BRM, BRG1, INI1, ARID1A,* and* PBRM1* 4, 5. The SWI/SNF complex plays an important role in the development of cancers 6-9. Loss of the SWI/SNF subunit can promote tumorigenesis. For example, loss of *INI1*, a tumor-suppressor gene, drives many malignant tumors 10, 11. More recently, 41% of clear cell RCCs were reported to have* PBRM1* mutations, and *PBRM1* is the second most commonly mutated gene in clear cell RCCs 12, 13.* ARID1A* mutation or deletion is found in 30% of endometrioid carcinomas and 50% of ovarian clear cell carcinomas 14, 15, 16.

*BRM*, a key subunit of the SWI/SNF chromatin remodeling complex, is an important mutant tumor suppressor gene in multiple tumors. *BRM* inactivation occurs in 10-20% of solid tumors, including lung cancer, prostate cancer, and gastric cancer, indicating that it plays an important role in multiple cancers 17, 18. In our previous study, BRM staining was absent in pure poorly differentiated RCCs and in poorly differentiated regions of composite RCCs 19. These results suggested that *BRM* may play an important role in tumor progression. However, we did not explore the oncogenic roles of *BRM* and the inactivation mechanism *in vitro* and *in vivo* in clear cell RCCs.

In this study, we found that copy number deletion and promoter hypermethylation contribute to the inactivation of *BRM,* and its overexpression can inhibit the viability of clear cell RCC cells* in vitro* and *in vivo*.

## Materials and Methods

### Cell culture

Human embryonic kidney 293T cells (293T), human clear cell RCC ACHN cells and 786-O cells were obtained from the Cell Bank of the Chinese Academy of Sciences (Shanghai, China). The cells were cultured in DMEM (Gibco, USA) containing 10% fetal bovine serum (Gibco), 100 units/ml penicillin, 100 μg/ml streptomycin, and 5.5 M MD-glucose (normal glucose) and incubated at 37°C in 5% CO_2_ and saturated humidity.

### Virus production and infection

Lipofectamine 2000 (Invitrogen, Shanghai, China) was used to transfect 293T cells with the packaging GV365 plasmid (Genechem, China), and the culture medium was replaced with complete medium at 8 h after transfection. After 48 h of culture in complete medium, the supernatant was collected, and the titer was determined by titration. Using the CON235 vector as the negative control, the *BRM* homozygous gene (NCBI GenBank accession no. NM_003070) was fused with a Flag-tag and then used to construct the *LV-BRM* overexpression vector. Clear cell RCC cells were seeded at a concentration of 3-5×10^4^ cells/well in complete medium followed by infection with the negative control lentivirus (NC) or overexpression lentivirus (OE). After overnight culturing, the medium was removed and replaced with normal medium. The infected cells were cultivated for another 72 h, and the stable clones were verified by inverted fluorescence microscopy and Western blotting analysis.

### Western blot analysis

The cells were washed three times with PBS, and total protein was isolated using protein lysis buffer. After centrifugation at 12,000 х g for 15 min at 4°C, cell debris was removed, and the supernatant (cell lysate) was used for Western blotting. Protein concentrations were measured using the BCA assay (Beyotime, Shanghai, China). Equal amounts of proteins were separated on 10% SDS-PAGE gels and then transferred onto PVDF membranes (Millipore, MA, USA). The membranes were blocked in blocking buffer (Tris-buffered saline, pH 7.6, 5% skim milk and 0.05% Tween) at room temperature for 1.5 h. Then, the membranes were incubated at 4°C overnight with a primary antibody diluted in blocking buffer followed by incubation with the corresponding secondary anti-IgG horseradish peroxidase conjugate (Santa Cruz Biotechnology, CA, USA) for 1.5 h. Antibody binding was visualized with ECL solution (Pierce Biotechnology, Inc., Rockford, IL, USA). The expression of BRM was assessed by immunoblotting using an antibody purchased from Abcam (ab15597, Abcam, Cambridge, MA, USA) and normalized to that of GAPDH.

### Analysis of apoptosis by flow cytometry

After 5 days of transfection, the cells were trypsinized and centrifuged at 12,000 х g for 5 min at 4°C. The cells were then washed in D-Hanks solution at 4 °C. Annexin V Apoptosis Detection Kit was purchased from eBioscience (cat. No. 88-8007).

### Cell migration and invasion assay

Migration and invasion assays were performed using transwell migration chambers (Corning, Cat# 354578) and Matrigel invasion chambers (Corning, Cat#354483), respectively. The control and transfected cells were seeded at a density of 4×10^4^ cells/well. A volume of 100 μl of cells was added to the upper chamber, while 600 μl of DMEM containing 10% FBS was added to the lower chamber and incubated at 37°C in 5% CO_2_. The cells attached to the upper surface of the membrane were removed with a cotton swab, and the cells on the underside were fixed, stained with Giemsa (Dingguobio, Shanghai, China) for 3-5 min, and counted (nine random fields) by two independent investigators. The results were normalized to the controls.

### CCK8 assay

Cell-counting assay kit (CCK8) was purchased from Dojindo (Japan). Briefly, at 2 h before each indicated time point, 10 μl of CCK-8 solution was added to each well on a plate containing 100 μl of DMEM. Then, the absorbance at 450 nm was recorded using a microplate absorbance reader. Each count was determined as an average of three replicates, and each data point was the average of at least three experiments. All data were normalized to the control group.

### Xenograft studies

Protocols for the research project were approved by the Ethics Committee of Nanjing General Hospital and conformed to provisions of the Declaration of Helsinki. Animals were maintained under pathogen-free conditions and given free access to both food and water in a temperature- and light-controlled animal facility with a 12-hour light/dark cycle, and the temperature was kept at 23±3°C with a relative humidity of 50%±10%. The animals were allowed to adapt to their food and environment for 1 week before starting the experiment. For subcutaneous xenografts, ACHN cells were infected with *LV-BRM* and subjected to puromycin selection (2 μg ml^-1^) in vitro. Then, 5×10^6^ ACHN cells suspended with 100 Matrigel Matrix (Corning, Cat#354248) was injected subcutaneously into the backs of 8 female NOD.CB17-Prkdcscid/J mice per group (6 weeks of age, purchased from Nanjing University). Mice were euthanized 6 weeks after implantation, and subcutaneous tumors were collected and submitted for histological examination. Tumor lengths and widths were measured, and the volume was calculated according to the formula (length×width^2^)/2.

### Statistical analysis

The multiomics data of The Cancer Genome Atlas (TCGA) were downloaded from cBioPortal for Cancer Genomics, including genomic data of 418 patients, copy number variation data of 525 patients, transcription data of 534 patients and prognostic information of 522 patients in Jun 2017 (http://www.cbioportal.org/tutorial.jsp). *BRM* gene expression data was analyzed for differential expression in tumor tissues versus adjacent normal tissues. Furthermore, the association between *BRM* expression and survival in all tumor tissue samples was analyzed by Cox regression. GO pathway analysis was performed on all genes that were significantly associated with *BRM* expression in 499 clear cell RCC human tissues (P_FDA_<0.01 and Person>0.3). All analyses were performed using R-3.3.0 software. For experimental data, continuous values were presented as the means ± SE and tested by ANOVA analyses. Error bars represent SE for all figures. A value of P*<*0.05 was deemed statistically significant. Statistical analyses were performed using GraphPad Prism 6.01.

## Results

### Molecular mechanism underlying the inactivation of *BRM*

**Figure 1:**
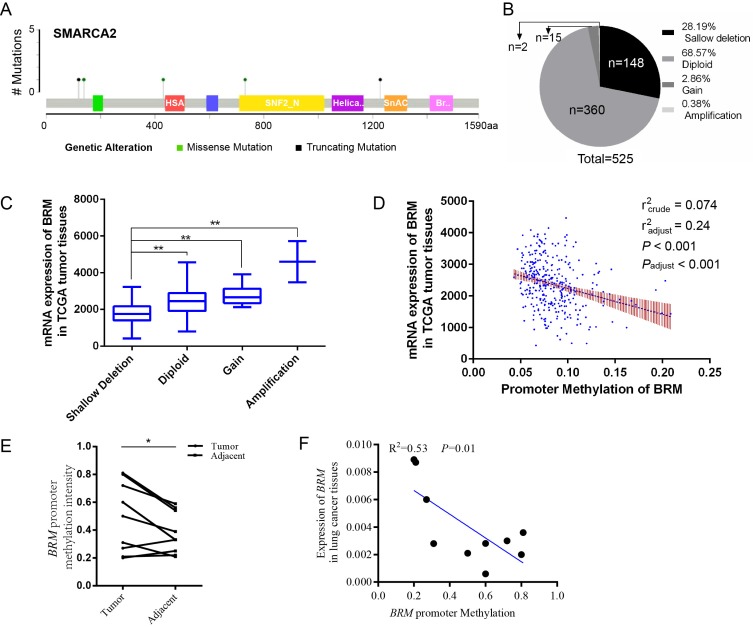
** Molecular mechanisms underlying *BRM* inactivation.** (A) Schematic diagram showing the positions of individual somatic alterations in the *BRM* gene identified in RCC. Truncating mutations (black) and missense mutations (black green) are displayed. (B and C) Copy number alterations of *BRM* in clear cell RCC samples from TCGA data and a box plot showing the association between mRNA levels and gene amplification or deletion. Data are expressed as the means ± SEM. ** *P* < 0.01. (D) The expression of *BRM* negatively correlates with *BRM* methylation in RCC patients (*P* < 0.001). The Spearman test was used with linear regression. The expression and methylation levels of *BRM* were downloaded from https://github.com/cBioPortal. The values are calculated by correlation analysis between the *BRM* read counts and methylation levels using linear regression. The band around the regression line indicates the 95% confidence interval of regression coefficients in multiple linear regression. Copy number variation was adjusted for the correlation analysis. Crude: No adjustment, adjust: adjusted by putative copy number variation. (E) Comparison of methylation intensity in tumor and adjacent tissues. The methylation status of CpG island in *BRM* promoter was analyzed by BSP in our own tissues, * *P* < 0.05, N=10/group. (F) Correlation of *BRM* expression and the promoter methylation intensity. The methylation status of CpG island in *BRM* promoter was analyzed by BSP in our own tissues, expression of *BRM* refers to *GAPDH*, N=10. The values are calculated by correlation analysis between the *BRM* read counts and methylation levels using linear regression.

In clear cell RCCs, we sought to elucidate the transcriptional regulation underlying low *BRM* gene expression. As shown in Figure 1A, somatic mutations, including truncating mutations and missense mutations, were presented in *BRM* in clear cell RCC patient samples. In the graphical representation of the somatic mutations in *BRM*, only one sample bears truncation mutation which might induce loss of function of *BRM*. Thus, we speculated that the inactivation of *BRM* is not primarily due to somatic mutations (mutation ratio 0.4%) (Figure 1A). Copy number alteration data revealed *BRM* gene deletions (28.19%) and diploid *BRM* genes (68.57%) in 525 cancer tissues (Figure 1B). Combined analysis of copy number alterations and gene expression data revealed that lower *BRM* mRNA levels were significantly associated with *BRM* deletions in renal tumor tissues (P<0.001) (Figure 1C). These results suggested that gene copy number deletion may be a mechanism underlying low *BRM* expression. Additionally, we found that the *BRM* promoter hypermethylation correlated with decreased expression of *BRM* (Figure 1D), implicating DNA hypermethylation as a novel mechanism for *BRM* inhibition (r_adjust_^2^=0.24, *P_adjust_*<0.001). To further confirm the above results, we validated our results in our own tissues. As a result, we found hypermethylation in tumors (*P* < 0.05), and the *BRM* expression was positively correlated with methylation levels (r^2^ = 0.53, *P* = 0.01, Figure 1E and 1F). Together, these findings suggested that copy number deletion and promoter hypermethylation were all important factors for the inactivation of *BRM* in clear cell RCC.

### BRM is absent in pure poorly differentiated RCCs, and low BRM expression is correlated with poorer overall survival

**Figure 2:**
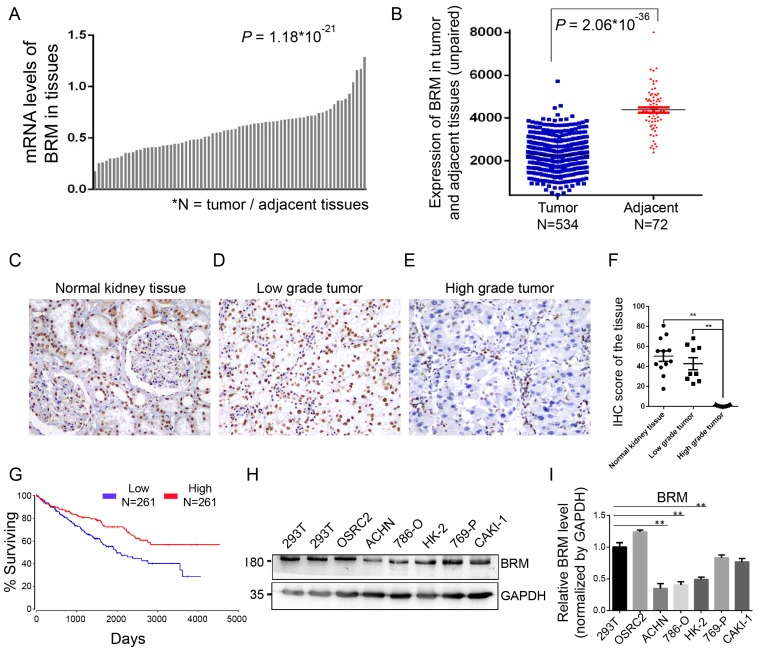
** BRM expression levels are lower in high-grade clear cell RCC tissues.** (A-B) Box plot showing lower levels of *BRM* mRNA in tumor tissues than in adjacent tissues. Data for the box plot were derived from the TCGA dataset (patient number = 534; *P* < 0.01). (C-E) BRM immunohistochemistry in low-grade tumor, high-grade tumor and normal tissues in clear cell RCC patient samples. Light brown staining shows BRM expression in scattered tumoral nuclei; negative nuclei are stained blue-purple. (F) Immunohistochemistry quantification using Image J. (G) Kaplan-Meier curve of the survival of patients with high and low levels of *BRM* mRNA (*P* < 0.05). The definitions of high and low expression are classified according to the median. The Kaplan-Meier curve indicates cancer-specific survival. (H) Western blotting to detect the levels of BRM in various RCC cell lines and human renal proximal tubular epithelial cells, HK-2. The changes in target protein levels are normalized to GAPDH levels. 293T cells were used as the positive control. (I) The results of Western blotting were confirmed by gray analysis using ImageJ software (n = 3). Data are expressed as the means ± SEM. ** *P* < 0.01.

To understand the involvement of *BRM* in clear cell RCC, we analyzed the mRNA expression of *BRM* in TCGA transcriptome data from 534 clear cell RCC patients. We found that the expression of *BRM* in tumor tissues was lower than adjacent tissues (Figure 2A and 2B). We also detected BRM in normal tissues, low-grade clear cell RCC and high-grade clear cell RCC by immunohistochemistry; significantly lower levels of BRM were observed in high-grade samples (Figure 2C-F). The corresponding Kaplan-Meier curve demonstrated that lower expression of *BRM* was significantly correlated with poor survival of clear cell RCC patients (Figure 2G). Next, we examined the levels of BRM protein in a panel of RCC cell lines, including 769-P, Caki-1, OSRC2, 786-O, ACHN, and the human renal proximal tubular epithelial cells HK-2 (Figure 2H and 2I). We observed significantly lower protein expression of BRM in the 786-O and ACHN cell lines compared to those in the others. Thus, 786-O and ACHN cell line were selected for the subsequent experiments.

### *BRM* inhibits the proliferation, migration and invasion of clear cell RCC cells

**Figure 3:**
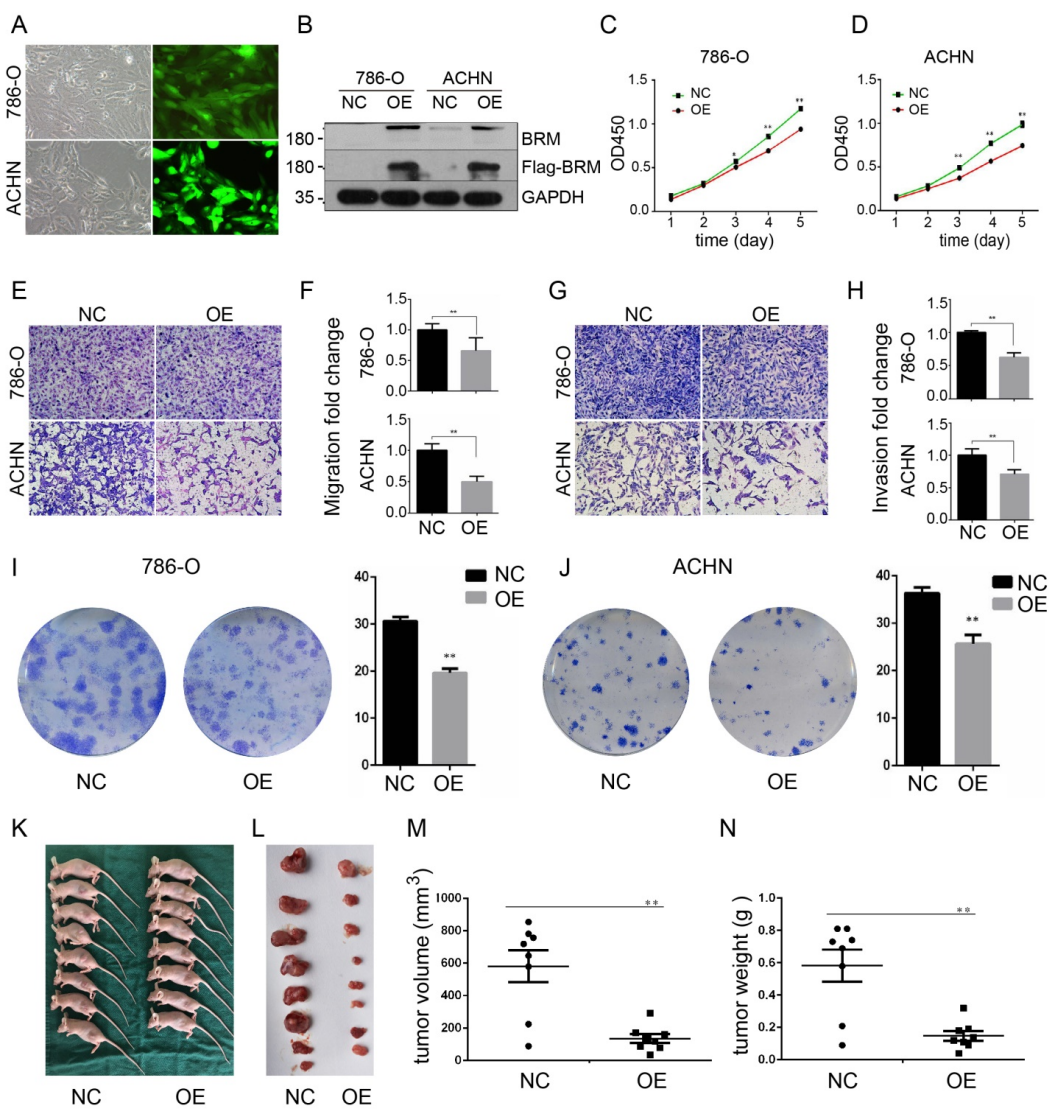
***BRM* inhibits the proliferation and suppresses the invasion and migration of clear cell RCC cells.** (A) Visualization of 786-O and ACHN cells in the OE group under a microscope. (B) Western blot analysis of BRM expression. (C) CCK8 assay in 786-O cells. (D) CCK8 assay in ACHN cells. (E-F) Migration assay performed in 786-O and ACHN cells stably overexpressing* BRM*. (G-H) The invasiveness of the *BRM*-overexpressing 786-O and ACHN cells as determined by the Matrigel invasion assay. (I-J) Plastic colony formation assay performed in 786-O and ACHN cells after *BRM* overexpression. Graphs are plotted from three independent experiments, and the error bar represents SEM. * *P* < 0.05, ** *P* < 0.01. (K-L) Representative nude mouse 6 weeks after injection of the indicated cells and Matrigel explants from each injected mouse (n = 8). (M-N) The subcutaneous tumor volume and weight of nude mice were measured after 6 weeks. Data are expressed as the means ± SEM. ** *P* < 0.01.

To further investigate the functions of *BRM* in clear cell RCC, we overexpressed *BRM* in 786-O and ACHN cells. The cells were divided into two groups, the OE group and the NC group. Control lentivirus and *BRM*-overexpressing lentivirus were transfected into the 786-O and ACHN cell lines. The cells were grown to 80% confluence, and GFP fluorescence was then observed under an inverted fluorescence microscope (Figure 3A). Western blot analysis was used to verify the overexpression of BRM (Figure 3B). The results of the CCK8 experiment also showed that overexpressing *BRM* inhibited the growth of ACHN and 786-O cells (Figure 3C and 3D). Furthermore, *BRM*-overexpressing 786-O and ACHN cells showed significant reductions in their abilities of migration (Figure 3E and 3F) and invasion (Figure 3G and 3H). *BRM*-overexpression cells showed reductions in both the number and size of individual colonies, which further signified the involvement of *BRM* in controlling the tumorigenic properties of clear cell RCC cells (Figure 3I and 3J). These results suggested the involvement of *BRM* in the tumorigenic and metastatic properties of clear cell RCC cells. To further examine the oncogenic activity of *BRM in vivo*, we subcutaneously injected 5×10^6^ transduced cells into nude mice. Recipient mice developed a subcutaneous mass at 80% penetrance. Notably, *BRM* virtually inhibited the xenograft tumor growth of ACHN cells (Figure 3K-N).

### *BRM* promotes apoptosis and cell cycle arrest

**Figure 4:**
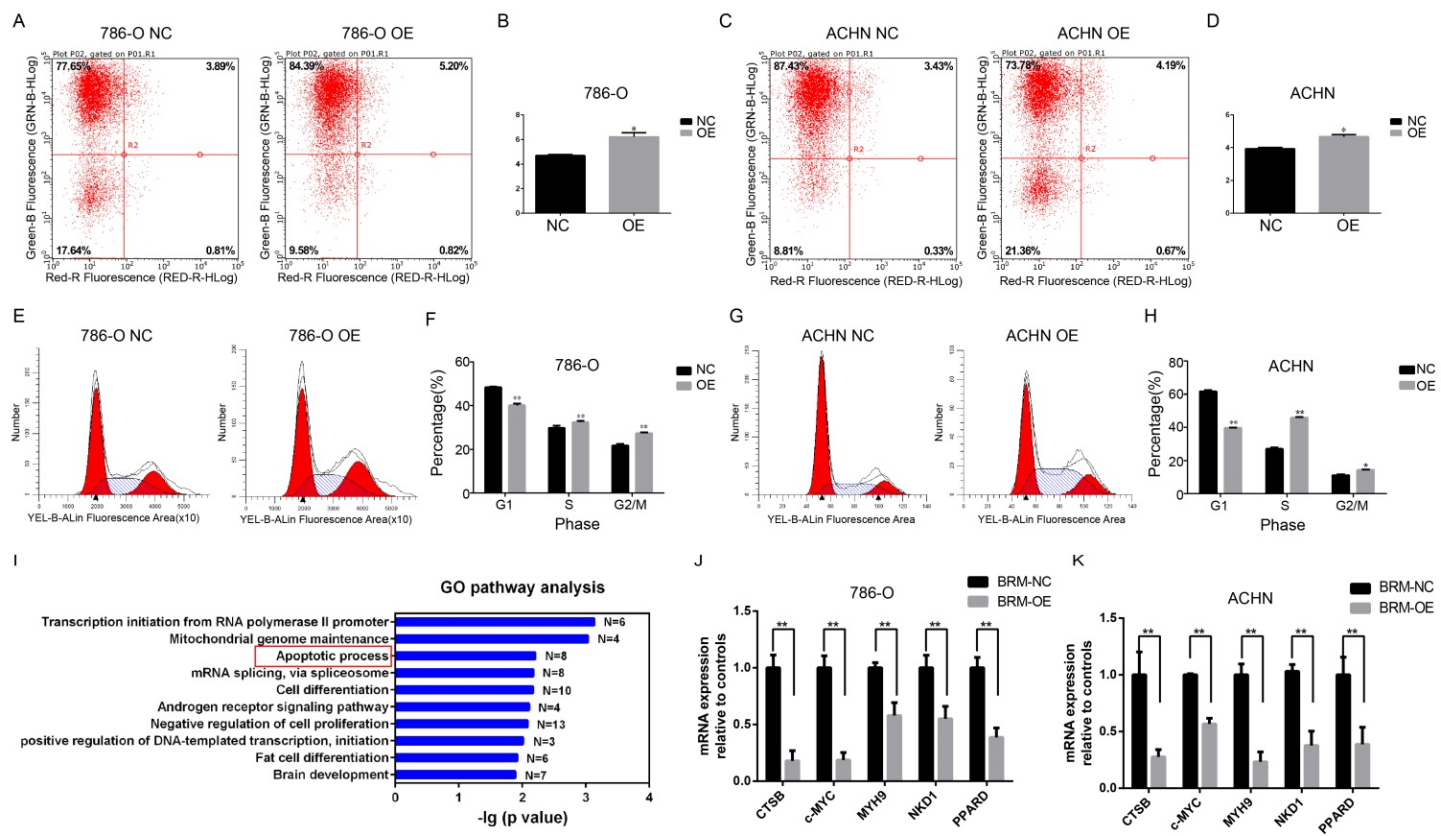
***BRM* promotes apoptosis and regulates apoptosis pathways in clear cell RCC cells.** Percentage of apoptotic 786-O (A-B) and ACHN (C-D) cells were detected by flow cytometry analysis. The lower right quadrant represents the early apoptotic cells, and the upper right quadrant represents the late apoptotic cells. (E-H) Proportions of cells at different cell cycle phases in the OE and NC groups. (I) GO pathway analysis of genes upregulated after *BRM* overexpression. The results were downloaded from https://github.com/cBioPortal. In total, 499 cases of clear cell RCC are included in the TCGA database. GO pathway analysis was performed on all genes that were significantly associated with *BRM* expression (*P*_FDA_<0.01 and Person>0.3). (J-K) The mRNA expression levels of anti-apoptotic genes in the OE and NC groups. All analyses were performed using R-3.3.0 software. Data are expressed as the means ± SEM. * *P* < 0.05, ** *P* < 0.01.

Meanwhile, flow cytometric analysis revealed that *BRM* overexpression in 786-O and ACHN cells enhanced apoptosis compared to that in the control group (Figure 4A-D). In addition, we examined the cell cycle profiles of 786-O and ACHN cells by fluorescence-assisted cell sorting. Compared to the NC group, the OE group had a significantly lower proportion of cells in the G1 phase and a higher proportion of cells in the S and G2/M phase (Figure 4E-H). These data indicated that *BRM* inhibited 786-O cell cycle progression. To further investigate mechanistic insights into the role of *BRM* in RCC, we involved all genes that were significantly correlated with *BRM* expression in TCGA tumor tissues (P_FDA_<0.01 and Person>0.3). Top ten of the significant GO terms were shown in Figure 4I. Consistent with our results, gene ontology analysis revealed that *BRM* plays an important role in apoptosis of cancers cells. The GO analysis of co-expressed genes in TGCA data is another hint to prompt us to investigate the roles of BRM in apoptosis. Thus we selected several anti-apoptotic genes, which were both previously reported to be regulated by BRM and correlated with BRM in TCGA data for further verification. Cell experiment further confirmed that *BRM* inhibited anti-apoptotic genes mRNA expression in 786-O and ACHN cells (Figure 4J-K).

## Discussion

The pathogenesis of clear cell RCC is related to inactivation of* VHL,* a well-known tumor suppressor gene 20-22. However, it was also reported that simple inactivation of the *VHL* gene does not directly lead to tumorigenesis. *VHL* gene inactivation is an early event in the development of clear cell RCC, and the driving development of clear cell RCC involves other oncogenic genes, such as the SWI/SNF family member *PBRM1* 3. It was also reported that the energy required for the SWI/SNF chromatin remodeling complex is derived from the catalytic subunits *BRM* and *BRG1* with ATPase activity 23-25. Initially, chromatin remodeling was thought to require intact complexes, but *BRM* has been found to produce chromatin remodeling activity in the absence of other subunits, which can be enhanced by the addition of other subunits 25. Altogether, these results indicated that the tumor suppressive ability of *BRM* is stronger than the other subunits.

Loss of BRM expression was observed in 40% of poorly differentiated clear cell RCCs 19, and BRM deletion occurred in only G4 grade RCC 12, 13, 26. Our previous studies showed that the lack of BRM was closely related to high tumor grades. Combined with the heterogeneity of BRM expression in composite tumors, it can be inferred that BRM may be a potential “second hit” candidate in clear cell RCC and play an important role in tumor progression. Further studies focusing on* BRM* are required to broaden our understanding of its involvement in tumor formation and progression. In our study, tumor tissues showed lower levels of *BRM* compared to the adjacent tissues. Besides, Kaplan-Meier curve demonstrated that lower expression was significantly correlated with reduced patient survival. Furthermore, we also found that *BRM* overexpression can inhibit the proliferation and migration of clear cell RCC *in vitro* and* in vivo*. Together, it was speculated that inactivation of *BRM* plays oncogenic roles in clear cell RCC.

In previous studies, the associations between the *BRM* promoter variants and cancers were observed and have potential therapeutic implications 27, 28. Thus further studies will be required to clarify the role of epigenetic *BRM* silencing as an oncogenic driver in the pathogenesis of cancers. To illustrate this mechanism underlying the inactivation of *BRM*, we used multiomics analysis of the TCGA database, and found that copy number deletion and promoter hypermethylation primarily contribute to the inactivation of *BRM* in clear cell RCC. These results were confirmed by our own cancer tissues. Furthermore, we also found that *BRM* overexpression can promote cell apoptosis and the cell cycle arrest in G2/M phase in vitro. GO analysis also revealed that *BRM* plays an important role in pathway of cell apoptosis. Arguably the most fundamental trait of cancer cells involves their ability to sustain chronic proliferation, which is resulted from the cell cycle deregulation 29, 30. Thus, we speculate that *BRM* may inhibit the proliferation of clear cell RCC by inducing G2/M phase arrest and cell apoptosis.

In conclusion, the present results indicate that the hypermethylation of *BRM* promoters plays oncogenic roles by the transcription inhibition of *BRM* in RCC, and the tumor suppressor gene *BRM* inhibits RCC cell vitality *in vitro* and* in vivo.* Besides, we confirmed that *BRM* may inhibit the proliferation of human clear cell RCC *in vitro* by inducing G2/M phase arrest and cell apoptosis.

## Abbreviations

RCC: renal cell carcinoma
*VHL*: von Hippel-Lindau
SWI/SNF: switch/sucrose nonfermenting
TCGA: The Cancer Genome Atlas
NC: negative control
OE: overexpression
